# Hypothermia for perinatal asphyxia: trial-based quality of life at 6–7 years

**DOI:** 10.1136/archdischild-2017-313733

**Published:** 2018-03-06

**Authors:** Helen Campbell, Oya Eddama, Denis Azzopardi, A David Edwards, Brenda Strohm, Oliver Rivero-Arias

**Affiliations:** 1 National Perinatal Epidemiology Unit (NPEU), Nuffield Department of Population Health, University of Oxford, Oxford, UK; 2 Centre for the Developing Brain, King’s College, London, UK; 3 Institute of Clinical Sciences, Imperial College, London, UK

**Keywords:** perinatal asphyxial encephalopathy, moderate hypothermia, health-related quality of life, long-term, health utilities index

## Abstract

**Objective:**

To assess the impact of hypothermic neural rescue at birth on health-related quality of life (HRQL) in middle childhood.

**Design:**

Six-year to 7-year follow-up of surviving children from the Total Body Hypothermia for Neonatal Encephalopathy (TOBY) Trial.

**Setting:**

Community study including a single parental questionnaire to collect information on children’s HRQL.

**Patients:**

145 children (70 in the control group, 75 in the hypothermia group) whose parents consented and returned the questionnaire.

**Interventions:**

Intensive care with cooling of the body to 33.5°C for 72 hours or intensive care alone.

**Main outcome measures:**

HRQL attributes and utility scores using the Health Utilities Index (HUI).

**Results:**

At 6–7 years, speech appeared disproportionately affected when compared with other aspects of HRQL but levels of normal emotional functioning were similar in both groups. The mean (SE) HUI3 HRQL scores were 0.73 (0.05) in the hypothermia group and 0.62 (0.06) in the control group; mean difference (95% CI) 0.11 (−0.04 to 0.26).

**Conclusions:**

Findings of non-significant differences were not unexpected; the study used data from long-term survivors in a neonatal trial and was underpowered. However, results favoured moderate hypothermia and so complement the clinical results of the TOBY Children study. The work provides further insight into the long-term HRQL impact of perinatal asphyxial encephalopathy and provides previously unavailable utility data with which to contemplate the longer term cost-effectiveness of hypothermic neural rescue.

**Trial registration number:**

This study reports on the follow-up of the TOBY clinical trial: ClinicalTrials.gov number NCT01092637.

**What is already known on this topic?**

- The benefits of hypothermic neural rescue in terms of cognitive and disability-free survival persist into middle childhood.
- Data on generic health-related quality of life (HRQL) utilities following hypothermia have not previously been reported.

**What this study adds?**

- New data on HRQL utility at 6–7 years after hypothermia plus intensive care and intensive care alone for perinatal asphyxia.
- Further insight into how different aspects of HRQL are affected following treatment for perinatal asphyxia.

## Introduction

Perinatal asphyxial encephalopathy is associated with a high risk of death or early neurodevelopmental impairment, and survivors frequently develop functional disabilities and cognitive impairments in later childhood.^1^ Evidence from randomised trials demonstrates that moderate hypothermia to 33°C–34°C for 72 hours initiated within 6 hours of delivery in infants with clear evidence of asphyxial encephalopathy reduces the risk of death or disability at 18–24 months of age and increases the rate of survival without disability.^2^

Since 2010, guidelines have recommended hypothermia for the treatment of moderate to severe perinatal asphyxial encephalopathy.^3–6^ Longer term follow-up of children in three major randomised trials of hypothermia (the Total Body Hypothermia for Neonatal Encephalopathy (TOBY) Trial, the CoolCap Trial and the National Institute of Child Health and Human Development (NICHD) Trial) has also confirmed that the clinical benefit at 18 months persists or is maintained, at least in part, into middle childhood.^7–9^

The question as to whether such clinical benefits translate into improvements in health-related quality of life (HRQL) for children is important. Parents want to know whether hypothermia can bring about long-term improvements in the overall well-being of their children. Healthcare professionals want to know what aspects of HRQL continue to be affected by perinatal asphyxial encephalopathy and hypothermia. Health economists need estimates of long-term generic HRQL to be able to contemplate cost-effectiveness. The CoolCap and NICHD Trials have reported various long-term data including those relating to the functional independence, cognitive outcomes and physical, emotional and social well-being of children.^7 8 10^ In this paper, we present the first comparative analysis of generic HRQL using data from the Health Utilities Index (HUI), which were collected during the 6-year to 7-year follow-up of surviving children in the TOBY Trial.^11^

## Methods

### Study population

Parents of surviving children who participated in the TOBY Trial were invited to take part in the TOBY Children Follow-up Study when their child reached 6–7 years of age.^9^ The findings of TOBY Children Follow-up Study are reported elsewhere in detail; however, in brief, the study reported that at 6–7 years and when compared with the control group, a higher number of children in the hypothermia group had survived with an IQ score ≥85, survived without neurological abnormalities and had significant reductions in the risk of cerebral palsy and of moderate or severe disability.^9^

As part of that study, parents received a postal questionnaire including the HRQL HUI,^11^ and questions about the use of healthcare services by their child over the previous 6 months (to be reported elsewhere). The study included centres from the UK, Sweden, Hungary, Finland and Israel, but UK centres contributed most to the sample used in the analysis.

### Child HRQL

Parents completed the HUI questionnaire on behalf of their children.^11^ HUI measures generic health status and has two different scoring systems: HUI3 and HUI2. HUI3 was used here and is the preferred measure as it provides a more detailed descriptive system. HUI3 includes questions on eight attributes (vision, hearing, speech, ambulation, dexterity, emotion, cognition and pain), whereas HUI2 contains six attributes (sensation, mobility, emotion, cognition, self-care and pain). HUI2 was used for a secondary analysis.

For each HUI attribute, respondents select one of a number of prespecified descriptions covering a range of functioning levels from best or normal (level 1) to most severe (level 3, 4, 5 or 6 depending on the attribute and scoring system). For example, level descriptors for the hearing attribute on the HUI3 range from ‘Able to hear what is said in a group conversation with at least three other people, without a hearing aid’ (level 1) to ‘Unable to hear at all’ (level 5). When taken together, responses for each attribute provide a health state description for an individual, and for both the HUI3 and HUI2, these descriptive systems can be converted into a single index utility score using algorithms developed from a sample of general population preferences for a subset of HUI health states.^12–15^ Scores lie on a scale where 0 is equivalent to dead and 1 is equivalent to perfect health. Negative scores are also permitted and indicate health states considered worse than death. HUI was developed in Canada and so utility conversion algorithms from that country are widely used.^12 13^ Algorithms for the HUI2 in the UK are also available.^14 15^ In this study, the Canadian HUI3 and HUI2 algorithms and the HUI2 UK algorithm were both used.

In addition to the single index utility score, disability categories for HUI3 can also be derived using prespecified criteria based on the functional levels within each attribute.^16^

### Statistical analysis

Baseline characteristics between groups were compared using the χ^2^ test for differences in proportions, the Mann-Whitney U test for median differences and the test for trend for ordered groups developed by Cuzick.^17^ This test was also used when comparing HUI3 and HUI2 attribute levels between trial arms.

Single index utility scores were summarised using per patient means and standard errors. When comparing trial arms, mean differences and 95% CIs were estimated. Utility data were left skewed but as parametric and non-parametric confidence intervals were similar, only parametric intervals are reported.^18^ Parental responses from all study countries were included.

Just over 5% of data were missing for utilities. Data were assumed to be missing at random, and multiple imputation (MI) using chained equations was used to impute missing values (see online supplementary appendix).^19 20^ Following imputation, Rubin’s rule was used to generate combined estimates of means and SEs across MI datasets.^21^ Analyses were conducted in Stata MP V.13.^22^

10.1136/archdischild-2017-313733.supp1 Supplementary file 1

## Results

### Study population

There were 229 TOBY Trial survivors at 6–7 years; of these, 184 (80%) parents/carers consented to participate in the main TOBY Children Study, and the remaining 45 were lost to follow-up. One hundred and forty-five of the 184 parents (79%) returned the postal questionnaire and form the sample for this study. One hundred and thirty of these 145 parents were from the UK (14 were from Hungary and 1 from Finland). Thirty-nine of 184 (21%) families either did not respond to or declined the questionnaire.

Seventy-five children were from the hypothermia arm and 70 from the control arm. There were no differences between arms in terms of baseline demographics or clinical characteristics at trial entry (table 1). At 6–7 years, a higher proportion of children in the hypothermia group had normal neurological function and an IQ score ≥85 and fewer had moderate or severe levels of disability.

**Table 1:** Child demographics, clinical characteristics and neurological function at 6–7 years by trial arm for surviving children with questionnaire data

	Control group (n=70)	Hypothermia group (n=75)	P value
Baseline demographics and characteristics at trial entry			
Male sex, n (%)	41 (59)	47 (63)	0.61
Missing	0	0	
Age (years)			
Median (IQR)	6.3 (6.1–6.7)	6.3 (6.1–6.8)	0.36
Missing	3	1	
Gestational age (weeks)			
Median (IQR)	40.1 (39–41)	40.2 (39.3–41.4)	0.48
Missing	10	7	
Birth weight (g)			
Median (IQR)	3400 (3200–3850)	3460 (3172–3828)	0.80
Missing	0	0	
Delivery complications, n (%)	52 (75)	57 (77)	0.82
Missing	1	1	
Apgar score ≤5 at 10 min, n (%)	42 (74)	41 (71)	0.72
Missing	13	17	
Outcomes at 6–7 years			
Normal neurological function, n (%)	35 (50)	50 (67)	0.04
Missing	0	0	
IQ ≥85, n (%)	43 (64)	59 (79)	0.06
Missing	3	0	
Overall disability*, n (%)			
None or mild	42 (60)	59 (81)	0.006
Moderate or severe	28 (40)	14 (19)	
Missing	0	2	

IQR Inter-quartile range; IQ Intelligence Quotient; *Overall disability: mild disability (an IQ score of 70–84, level 1 gross motor function (is able to walk independently but may have some gait abnormalities), or abnormality in one or both eyes with normal or nearly normal vision); moderate disability (an IQ score of 55–69, level 2 or 3 gross motor function (has minimal ability to perform gross motor skills or requires assistance with walking) or moderately reduced vision); severe disability (an IQ score of <55, level 4 or 5 gross motor function (needs adaptive seating or has severely limited mobility) or no useful vision).

A comparison of the 145 included children and the 39 non-participants revealed no significant differences (see online supplementary appendix table A1). Also, among the 39 non-participating families (and as observed for the participating families), a higher proportion of children in the hypothermia group than in the control group had an IQ ≥85 (16/23 (70%) vs 9/16 (56%), P=0.394) and had normal neurological functioning (14/23 (61%) vs 6/16 (38%), P=0.151).

When compared with the surviving children for whom primary outcome data were available at 6–7 years, the 45 children who were lost to follow-up had a higher frequency (although not significantly so) of severe abnormalities on amplitude-integrated electroencephalogram (EEG) at trial entry and lower scores on the Mental Development Index at 18 months. Eighteen of these 45 children (40%) were in the hypothermia arm and 27 (60%) were in the control arm.^9^

### Child HRQL

Table 2 shows the attribute levels recorded on the HUI3 (those for the HUI2 are shown in online supplementary appendix table A2). For each attribute, the proportion of children reported by their parents as functioning at level 1 was greater in the hypothermia group than in the control group, although not significantly so. Differences were particularly pronounced for the speech (74% vs 59%) and dexterity (76% vs 64%) attributes. The smallest difference was seen on the emotion attribute, with 85% of parents in the hypothermia arm and 84% in the control arm considering their children to be functioning at normal emotional levels.

**Table 2:** Distribution of responses (n, (%)) for each of the HUI3 attributes by trial arm

Level	Vision		Hearing		Speech		Emotion		Pain		Ambulation		Dexterity		Cognition	
	Control n=70	Hypoth. n=75	Control n=70	Hypoth. n=75	Control n=70	Hypoth. n=75	Control n=70	Hypoth. n=75	Control n=70	Hypoth. n=75	Control n=70	Hypoth. n=75	Control n=70	Hypoth. n=75	Control n=70	Hypoth. n=75
1	56 (81.16)	64 (85.33)	63 (94.03)	71 (98.61)	41 (58.57)	55 (74.32)	59 (84.29)	63 (85.14)	54 (77.14)	61 (82.43)	48 (68.57)	59 (78.67)	45 (64.29)	57 (76.00)	45 (67.16)	53 (71.62)
2	7 (10.14)	8 (10.67)	3 (4.48)	0	9 (12.86)	2 (2.70)	6 (8.57)	11 (14.86)	7 (10.00)	8 (10.81)	2 (2.86)	2 (2.67)	2 (2.86)	2 (2.67)	6 (8.96)	6 (8.11)
3	1 (1.45)	1 (1.33)	1 (1.49)	1 (1.39)	7 (10.00)	7 (9.46)	2 (2.86)	0	6 (8.57)	3 (4.05)	2 (2.86)	0	3 (4.29)	0	2 (2.99)	3 (4.05)
4	0	0	0	0	3 (4.29)	2 (2.70)	1 (1.43)	0	2 (2.86)	1 (1.35)	5 (7.14)	4 (5.33)	4 (5.71)	7 (9.33)	4 (5.97)	2 (2.70)
5	2 (2.90)	1 (1.33)	0	0	10 (14.29)	8 (10.81)	2 (2.86)	0	1 (1.43)	1 (1.35)	2 (2.86)	2 (2.67)	3 (4.29)	3 (4.00)	4 (5.97)	5 (6.76)
6	3 (4.35)	1 (1.33)	0	0	–	–	–	–	–	–	11 (15.71)	8 (10.67)	13 (18.57)	6 (8.00)	6 (8.96)	5 (6.76)
Missing	1	0	3	3	0	1	0	1	0	1	0	0	0	0	3	1
Test for trend	0.76, P=0.446		1.42, P=0.154		1.72, P=0.086		0.32, P=0.751		0.88, P=0.380		1.34, P=0.181		1.66, P=0.096		0.60, P=0.551	

Descriptive labels for level 1 on each attribute are as follows:

Vision: able to see well enough to read ordinary newsprint and recognise a friend on the other side of the street, without glasses or contact lenses.

Hearing: able to hear what is said in a group conversation with at least three other people, without a hearing aid.

Speech: able to be understood completely when speaking with strangers or friends.

Emotion: happy and interested in life.

Pain: free of pain and discomfort.

Ambulation: able to walk around the neighbourhood without difficulty and without walking equipment.

Dexterity: full use of 2 hands and 10 fingers.

Cognition: able to remember most things, think clearly and solve day-to-day problems.

Hypoth., Hypothermia group.

Table 3 shows the HUI3 and HUI2 HRQL utility scores, by trial arm and for complete case and MI analyses. Following imputation, utility scores were universally lower. This is because children with missing data were less likely to have normal neurological function and an IQ ≥85 and were more likely to have multiple handicaps and moderate or severe levels of disability. Table 3 shows that for both analyses and for any utility conversion algorithm used, children in the hypothermia group, on average, presented with higher utility scores than children in the control group, although not significantly so.

**Table 3:** HUI3 and HUI2 HRQL utility scores, by trial arm and for complete case and multiple imputation analyses

HUI version	Control group (n=70)					Hypothermia group (n=75)					Mean difference (95% CI)
	n	Missing	Mean	SD	SE	n	Missing	Mean	SD	SE	
Multiple imputation analysis											
HUI3 score (Canadian valuation)	70	0	0.618	–	0.058	75	0	0.730	–	0.049	0.112 (−0.038 to 0.262)
HUI2 score (Canadian valuation)	70	0	0.744	–	0.037	75	0	0.821	–	0.032	0.078 (−0.019 to 0.174)
HUI2 score (UK valuation)	70	0	0.733	–	0.038	75	0	0.809	–	0.032	0.076 (−0.021 to 0.174)
Complete case analysis*											
HUI3 score (Canadian valuation)	66	4	0.658	0.456	0.056	72	3	0.746	0.409	0.048	0.089 (−0.057 to 0.234)
HUI2 score (Canadian valuation)	66	4	0.772	0.292	0.036	71	4	0.829	0.266	0.032	0.057 (−0.038 to 0.151)
HUI2 score (UK valuation)	66	4	0.763	0.290	0.036	71	4	0.821	0.263	0.031	0.058 (−0.035 to 0.152)

SD standard deviation; SE standard error; CI parametric confidence intervals; *Children for whom an HUI utility score was calculable from questionnaire responses.

HRQL, health-related quality of life; HUI, Health Utilities Index.

The associated disability categories for the HUI3 are shown in table 4 and similarly show lower levels of disability (although not significantly so) in children randomised to hypothermia.

**Table 4:** HUI3 disability categories* by trial arm

Disability category	Control group n (%)	Hypothermia group n (%)
1: No disability	26 (39)	39 (54)
2: Mild disability	10 (15)	8 (11)
3: Moderate disability	6 (9)	7 (10)
4: Severe disability	24 (36)	18 (25)
Total	66 (100)	72 (100)
Missing	4	3
Test for trend	1.72, P=0.085	

Disability categories described as follows:

Level 1: no disability or perfect health. The associated utility score value is 1.00, and all attributes (dimensions or domains) of health status are at their highest functional level (level 1).

Level 2: mild disability. The associated utility scores range from 0.89 to 0.99, and at least one attribute is at reduced level of function that can be readily corrected and/or does not prevent any activities.

Level 3: moderate disability. The associated utility score ranges from 0.70 to 0.88, and at least one attribute is at reduced level of function that cannot be corrected and/or prevents some activities.

Level 4: severe disability. The associated utility score is less than 0.70, and at least one attribute is at reduced level of function that cannot be corrected and prevents many activities.

*Category labels and descriptions taken from: Feng *et al*.^29^

HUI, Health Utilities Index.

## Discussion

This study is the first to offer a comparative assessment of HRQL using the HUI for children 6–7 years after randomisation to standard care with hypothermia or standard care alone for perinatal asphyxia. The HUI is a leading measure of generic HRQL and provides rich information on levels of functioning for key health-related attributes.^11^ It also facilitates the calculation of single index utility scores that summarise a subject’s health status relative to the anchors of death and perfect health and that can be used to estimate quality-adjusted life years for use in cost-effectiveness analyses.^11^

The data presented here showed that for each individual HUI3 attribute, more parents in the hypothermia arm than in the control arm categorised their child as functioning at normal age appropriate levels (level 1) and that these differences fed through into higher average utility scores and lower disability levels for children randomised to hypothermia. None of the differences we observed however achieved statistical significance, and this was perhaps unsurprising because as the follow-up of a randomised trial and using a sample size fixed from the original trial, we were underpowered to detect such significant differences (an ex-postanalysis of power confirmed this). In addition, we cannot completely exclude the role of chance in our results. However, and when considered alongside the main clinical outcomes from the TOBY Children Study, the direction of the HRQL figures in favour of hypothermia appear intuitive.^9^

Differences in attribute levels between trial arms were particularly pronounced for the speech domain (table 2), with 74% and 59% of parents in the hypothermia and control groups, respectively, reporting their child to be functioning at normal levels (level 1). Also apparent is that the proportions of children categorised as normal on the speech attribute are noticeably lower than the proportions categorised as normal on the other attributes, suggesting that in general, for these children, speech may be disproportionately affected as compared with other aspects of HRQL. A similar finding has been observed elsewhere. The NICHD follow-up study assessed various measures of intelligence for children 6–7 years after perinatal asphyxia and reported that verbal IQ scores generally showed greater deficits when compared with other measures such as performance IQ and processing speed quotients.^10^

Despite a greater proportion of children in the control arm having moderate to severe disabilities and abnormal neurological functioning, 84% were still reported as being ‘happy and interested in life’ (level 1) on the HUI3 emotion attribute as compared with 85% in the hypothermia arm. The similarity of these figures suggests that the level of disability faced by these children may not necessarily impact on their ability to be happy. In the NICHD follow-up study, parents also reported on their child’s emotional functioning at 6–7 years using the Child Health Questionnaire (CHQ).^23^ Similar proportions in the hypothermia and control arms (75% vs 76%) reported they were ‘very satisfied’ with their child’s level of self-esteem.^8^ Published research in children with cerebral palsy has similarly shown emotional functioning appears to be unrelated to the severity of the condition.^24^ From parent-proxy responses on the HUI3, Kennes *et al* 24 showed correspondingly high proportions of children reported as being ‘happy and interested in life’ across five cerebral palsy severity categories. These findings are notable and may help parents of babies with disabilities as a consequence of perinatal asphyxia, who may anticipate that their child will suffer in the longer term. One must however acknowledge that these findings come from parent-proxy responses rather than directly from the children per se.

The utility scores computed using the HUI3 system were lower than those estimated using HUI2 (table 3), a finding also observed elsewhere and which is likely attributable to the HUI3 having more severity levels per attribute and therefore facilitating a greater range for states describing severe impairment.^25^ Regardless of the system used, the difference between trial arms in perceived HRQL favoured hypothermia and is likely to be large enough to be important to the individuals involved. The developers of the HUI have reported that differences of 0.05 or greater are meaningful, and smaller differences might well be too.^11^ As alluded to above, the larger utility scores observed with hypothermia complement the clinical results of the TOBY Children Study and suggest that the objective assessment of neurological function is mirrored in a sense of overall well-being.^9^ However, and also worthy of note, is that utility scores for children in this study were lower than normative values (0.93) that have been recorded for children in the general population.^26 27^

Like the TOBY Children Study, the CoolCap and NICHD follow-up studies also used parents as proxy respondents, although neither used the HUI.^7 8^ The CoolCap study used the WeeFIM (Functional Independence Measure for Children) questionnaire, which includes cognition, mobility and self-care domains like the HUI; however, the range of items included in each attribute and how scores are calculated is different, making direct comparisons difficult.^7 28^ In the NICHD follow-up study, parents completed the CHQ, rating physical health and self-esteem.^8 23^ Although not directly comparable with the HUI3 ambulatory and HUI2 mobility domains that here showed a greater proportion of children in the hypothermia group to have normal ability (table 2 and online supplementary appendix table A2), the physical health domain on the CHQ also showed a greater proportion of children in the hypothermia group, rated as having ‘excellent’ physical health at 6–7 years.

This study has a number of limitations. Questionnaire responses were unavailable for around 20% of children (39/184) whose parents consented to the TOBY Children Study. Analyses revealed no significant differences between responders and non-responders and also showed that when looking between trial arms at differences in clinical outcomes, the direction and magnitude of the differences observed were not dissimilar for both responding and non-responding families. Had these children been included in the analysis, one might hypothesise that the findings would not have altered substantially.

It is also necessary to contemplate the potential implications for our findings of the 45/229 (20%) surviving TOBY children who were lost to follow-up. Analyses showed these children to have had more severe abnormalities on EEG at trial entry and lower scores on the Mental Development Index at 18 months than children for whom follow-up data were available.^9^ With two-fifths of these 45 children in the hypothermia arm and three-fifths in the control arm, had they been included in the analysis, then the potential exists for a lowering of the absolute utility scores observed here and for an increase in the difference in utility between the trial arms in favour of hypothermia.

Finally, the issue of missing data must be acknowledged; although, in this study, the amount of missing data was small at just 5%. Nevertheless, the mean difference favouring hypothermia increased following imputation (table 3). Of the children with missing utility data, more in the control arm than in the hypothermia arm had moderate or severe levels of disability (4/4 vs 2/4) and multiple disabilities (4/4 vs 2/4), which likely translated into lower imputed levels of utility.

## Conclusions

This study is the first to report comprehensive HRQL using the HUI in children surviving 6–7 years following randomisation to hypothermia or standard care alone for perinatal asphyxia. Non-significant differences in HRQL favouring moderate hypothermia corroborate the clinical findings of the TOBY Children Study. This study also provides previously unavailable data of interest to parents, health policy makers and health economists who may now wish to re-evaluate the long-term cost-effectiveness of hypothermia for perinatal asphyxia.
