# Supplementary material for: Hypothermia for perinatal asphyxia: trial-based quality of life at 6–7 years
Source: Arch Dis Child. 2018 Mar 6;103(7):654–9. doi: 10.1136/archdischild-2017-313733 (PMC6047162; doi:10.1136/archdischild-2017-313733)
Supplement: Supplementary file 1 [file archdischild-2017-313733supp001.docx]

**Online Web Appendix**

**Missing Data and Multiple Imputation**

Multiple imputation utilises regression-based approaches to predict a number of different values for each missing data point, in essence creating several different datasets.^1 2^ The approach retains the correlation structure between variables and by imputing numerous values explicitly accounts for uncertainty not only within the prediction model parameters (i.e. that they are derived only from a sample of data) but also for stochastic variability (i.e. that patients with the same values of covariates within a prediction model may not necessarily have the same outcome).

Here, fifty values were imputed for each missing data point, and imputation was performed for healthcare contact data (results to be reported elsewhere) and HUI utility score data simultaneously by specifying separate regression models for each variable with missing data. Within each model, the remaining variables with missing data (HUI utility and healthcare contact variables) were used as predictors along with variables having complete data upon trial entry (e.g. gender, birthweight, head circumference) and study clinical endpoints at 6-7 years (e.g. disability level and IQ ≥ 85). Imputation was performed using prediction mean matching, and for each trial arm separately.

**References**

1. White IR, Royston P, Wood AM. Multiple imputation using chained equations: Issues and guidance for practice. *Stat Med* 2011;30(4):377-99. doi: 10.1002/sim.4067 [published Online First: 2011/01/13]

2. Faria R, Gomes M, Epstein D, et al. A guide to handling missing data in cost-effectiveness analysis conducted within randomised controlled trials. *PharmacoEconomics* 2014;32(12):1157-70. doi: 10.1007/s40273-014-0193-3

**Results**

Table A1 below compares the characteristics and 6-7 year outcomes for surviving children whose parents completed the follow-up questionnaire and for surviving children whose parents did not respond or who declined the questionnaire. No significant differences between non-participants (n=39) and participants (n=145) were detected.

**Table A1: Baseline demographics and selected clinical characteristics at trial entry at 6-7 years, for sub-samples of children with returned and missing parent questionnaires**

|  | **Returned parent questionnaire** | **Missing parent questionnaire** | **p-value** |
| --- | --- | --- | --- |
|  | **n=145** | **n=39** |  |
| *Trial allocation* |  |  |  |
| Control Group | 70 (48) | 16 (41)* |  |
| Hypothermia Group | 75 (52) | 23 (59)* |  |
| **Baseline demographics and characteristics at trial entry** | | |  |
| *Male sex, n (%):* | 88 (61) | 25 (64) | 0.70 |
| Missing | 0 | 0 |  |
| *Gestational age (weeks):* |  |  |  |
| Median (IQR) | 40.1 (39.1-41.1) | 40.6 (39-41.3) | 0.70 |
| Missing | 17 | 6 |  |
| *Birth weight (grams):* |  |  |  |
| Median (IQR) | 3446 (3190-3828) | 3500 (3160-3950) | 0.92 |
| Missing | 0 | 0 |  |
| *Delivery complications, n (%):* | 109 (76) | 25 (66) | 0.19 |
| Missing | 2 | 1 |  |
| *Apgar score ≤5 at 10 minutes, n (%):* | 83 (72) | 27 (79) | 0.40 |
| Missing | 30 | 5 |  |
| **Outcomes at 6-7 years** | | |  |
| *Normal neurological function, n (%):* | 85 (59) | 20 (51) | 0.41 |
| Missing | 0 | 0 |  |
| *IQ ≥ 85, n (%)* | 102 (72) | 25 (64) | 0.35 |
| Missing | 3 | 0 |  |
| *Overall disability†, n (%):* |  |  |  |
| None or mild | 101 (71) | 26 (67) | 0.63 |
| Moderate or severe | 42 (29) | 13 (33) |  |
| Missing | 2 | 0 |  |

IQR Inter-quartile range; IQ Intelligence Quotient;

* Amongst the 39 non-participating families, a higher proportion of children in the hypothermia group than in the control group had an IQ≥85 (16/23 (70%) v 9/16 (56%), p=0.394) and had normal neurological functioning (14/23 (61%) v 6/16 (38%), p=0.151).

†Overall disability - mild disability (an IQ score of 70 to 84, level 1 gross motor function [is able to walk independently but may have some gait abnormalities], or abnormality in one or both eyes with normal

or nearly normal vision); moderate disability - (an IQ score of 55 to 69, level 2 or 3 gross motor function [has minimal ability to perform gross motor skills or requires assistance with walking], or moderately reduced vision); severe disability (an IQ score of <55, level 4 or 5 gross motor function [needs adaptive seating or has severely limited mobility], or no useful vision)

Table A2 below shows the levels reported for the HUI2 attributes. A similar pattern to that observed for the HUI3 attributes was also seen for the HUI2, with the largest difference seen on the sensation attribute (52% v 68% for level 1) which includes a speech component. The proportion of children considered by their parents to be functioning at a normal emotional level was also remarkably similar across both trial arms (74% v 76%). Again, however, none of the differences achieved statistical significance.

**Table A2: Distributions of responses (n, (%)) for each of the HUI2 attributes, by trial arm**

| **Level** | **Sensation** | | **Mobility** | | **Emotion** | | **Cognition** | | **Self-care** | | **Pain** | |
| --- | --- | --- | --- | --- | --- | --- | --- | --- | --- | --- | --- | --- |
|  | **Control n=70** | **Hypoth.**  **n=75** | **Control n=70** | **Hypoth.**  **n=75** | **Control n=70** | **Hypoth.**  **n=75** | **Control n=70** | **Hypoth.**  **n=75** | **Control n=70** | **Hypoth.**  **n=75** | **Control n=70** | **Hypoth.**  **n=75** |
| 1 | 36  (52.17) | 50  (67.57) | 48  (68.57) | 59  (78.67) | 52  (74.29) | 55  (76.39) | 45  (67.16) | 53  (71.62) | 43  (61.43) | 55  (73.33) | 55  (79.71) | 62  (84.93) |
| 2 | 5  (7.25) | 4  (5.41) | 2  (2.86) | 2  (2.67) | 12  (17.14) | 14  (19.44) | 12  (17.91) | 11  (14.86) | 5  (7.14) | 3  (4.00) | 10  (14.49) | 8  (10.96) |
| 3 | 16  (23.19) | 12  (16.22) | 7  (10.00) | 4  (5.33) | 4  (5.71) | 3  (4.17) | 4  (5.97) | 5  (6.76) | 1  (1.43) | 0 | 2  (2.90) | 3  (4.11) |
| 4 | 12  (17.39) | 8  (10.81) | 3  (4.29) | 4  (5.33) | 1  (1.43) | 0 | 6  (8.96) | 5  (6.76) | 21  (30.00) | 17  (22.67) | 2  (2.90) | 0 |
| 5 | - | - | 10  (14.29) | 6  (8.00) | 1  (1.43) | 0 | - | - | - | - | 0 | 0 |
| 6 | - | - | - | - | - | - | - | - | - | - | - | - |
| Missing | 1 | 1 | 0 | 0 | 0 | 3 | 3 | 1 | 0 | 0 | 1 | 2 |
| Test for trend | 1.87, p=0.061 | | 1.40, p=0.162 | | 0.43, p=0.664 | | 0.56, p=0.574 | | 1.42, p=0.156 | | 0.84, p=0.404 | |

Hypoth. – Hypothermia group

Descriptive labels for level 1 on each attribute are as follows:

Sensation: Able to see, hear, and speak normally for age.

Mobility: Able to walk, bend, lift, jump, and run normally for age.

Emotion: Generally happy and free from worry.

Cognition: Learns and remembers school work normally for age.

Self-care: Eats, bathes, dresses, and uses the toilet normally for age.

Pain: Free of pain and discomfort.
